# Endogenous *n*-3 Polyunsaturated Fatty Acids Are Beneficial to Dampen CD8^+^ T Cell-Mediated Inflammatory Response upon the Viral Infection in Mice

**DOI:** 10.3390/ijms20184510

**Published:** 2019-09-12

**Authors:** Kyung Won Kang, Seyoung Kim, Yong-Bin Cho, Seung Rok Ryu, Young-Jin Seo, Sang-Myeong Lee

**Affiliations:** 1 Division of Biotechnology, College of Environmental and Bioresources, Chonbuk National University, Iksan 54596, Korea; gp1900@jbnu.ac.kr (K.W.K.); rsr1894@jbnu.ac.kr (S.R.R.); 2 Department of Life Science, Chung-Ang University, Seoul 06974, Korea; sy121@cau.ac.kr (S.K.); dydqls1218@cau.ac.kr (Y.-B.C.)

**Keywords:** omega-3, CD8^+^ T cell, LCMV, immunopathology

## Abstract

Omega-3 (*n*-3) polyunsaturated fatty acids (PUFAs) have been known to exert anti-inflammatory effects on various disease states. However, its effect on CD8^+^ T cell-mediated immunopathology upon viral infection has not been well elucidated yet. In this study, we investigated the possible implication of *n*-3 PUFAs in CD8^+^ T cell responses against an acute viral infection. Infection of FAT-1 transgenic mice that are capable of synthesizing *n*-3 PUFAs from *n*-6 PUFAs with lymphocytic choriomeningitis virus (LCMV) resulted in significant reduction of anti-viral CD8^+^ T cell responses. Interestingly, expansion of adoptively transferred wild-type (WT) LCMV-specific T cell receptor (TCR) transgenic CD8^+^ (P14) T cells into FAT-1 mice was significantly decreased. Also, activation of anti-viral CD4^+^ helper T cells was reduced in FAT-1 mice. Importantly, P14 cells carrying the *fat-1* gene that were adoptively transferred into WT mice exhibited a substantially decreased ability to proliferate and produce cytokines against LCMV infection. Together, *n*-3 PUFAs attenuated anti-viral CD8^+^ T cell responses against an acute viral infection and thus could be used to alleviate immunopathology mediated by the viral infection.

## 1. Introduction

Polyunsaturated fatty acids (PUFAs) are fatty acids that contain two or more double bonds within their carbon backbone. The two major classes of PUFAs are omega-3 (*n*-3) and omega-6 (*n*-6) fatty acids, and they are known to affect human health and diverse disease conditions [1,2]. The FAT-1 transgenic mouse model, which expresses an *n*-3 PUFA desaturase that converts *n*-6 to *n*-3 fatty acids, has been widely used in numerous studies because *n*-6/*n*-3 PUFA profiles in this mouse are comparable to those supplied from dietary sources [3,4,5,6]. Importantly, the *n*-3 PUFAs have been known to exert anti-inflammatory effects on various inflammatory diseases including asthma [6,7], inflammatory bowel disease [8,9], and rheumatoid arthritis [10,11]. These immunosuppressive effects of *n*-3 PUFAs also have been often related with increased morbidity or mortality to various bacterial infections including *Listeria monocytogens*, *Mycobacterium tuberculosis*, and *Pseudomonas aeruginosa* in mice supplemented with dietary *n*-3 PUFAs or FAT-1 mice [12,13,14,15]. While numerous studies have been performed in bacterial infection models, there are only few studies have been reported in viral infection. Mice fed a diet enriched with *n*-3 PUFA were more susceptible to influenza virus infection, resulted in a higher viral load and mortality [16]. Another study demonstrated that either a dietary *n*-3 or *n*-6 PUFA did not affect mortality in mice infected with vaccinia virus [17]. Therefore, its role in anti-viral immune responses is still unclear and needed to be investigated further in different types of viral infections with advanced immunological approaches.

CD8^+^ T cells are major immune effectors that eliminate many viruses by killing infected cells [18,19,20], and they often rapidly produce inflammatory cytokines such as TNF-α and granzyme B, which are directly associated with immunopathology [21,22]. However, excessive activation of CD8^+^ T cells can result in severe immunopathology and often the death of virally infected hosts. For example, lethal lung injury can be caused by anti-viral CD8^+^ T cells upon influenza virus infection [23]. Also, anti-viral CD8^+^ T cells are known to be major contributors to lethal Ebola virus infections [24]. Therefore, attenuating excessive anti-viral CD8^+^ T cell responses would be beneficial to alleviate unwarranted immunopathology.

Lymphocytic choriomeningitis virus (LCMV) is a single-strand RNA virus belonging to the family *Arenaviridae*, whose natural host is the mouse [25]. While the clone 13 strain of LCMV causes persistent infection, the Armstrong strain causes an acute infection where viruses are rapidly eradicated [26,27]. Infection of the mouse with LCMV Armstrong triggers robust CD8^+^ T cell responses, which results in the rapid elimination of viruses within 7 to 8 days post-infection [27,28,29]. Therefore, the infection of laboratory mice with LCMV Armstrong is a widely used model to study mechanisms of anti-viral CD8^+^ T cell responses.

To gain insights into the role of *n*-3 PUFAs in anti-viral T cell responses, in this study, FAT-1 transgenic mice were infected with an acute strain of LCMV. In addition, P14 cells carrying *fat-1* gene were used to obtain direct evidence of involvement of *n*-3 PUFAs in anti-viral CD8^+^ T cell responses during an acute viral infection.

## 2. Results

### 2.1. The Expansion of Splenocytes in FAT-1 Mice Was Limited upon Acute LCMV Infection

**Figure 1:**
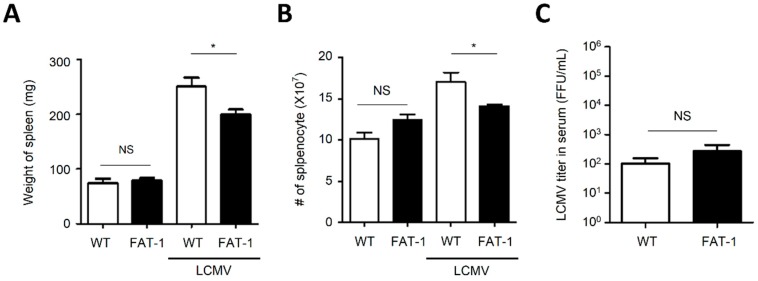
The expansion of splenocytes from FAT-1 mice was less than that of wild type (WT) mice upon infection with lymphocytic choriomeningitis virus (LCMV). WT and FAT-1 mice were infected with 2 × 10^5^ focus-forming unit (FFU) of LCMV (Armstrong) for 7 days. (**A**) The weight of spleens from WT and FAT-1 mice were measured. (**B**) The total number (#) of splenocytes from WT and FAT-1 mice were counted. (**C**) Viral titers in the serum of WT and FAT-1 mice were determined by focus-forming assays. The data represent the mean ± SEM. * *p* ≤ 0.05; NS, not significant. The data represent the results of at least three independent experiments.

To investigate the possible implication of *n*-3 PUFAs in anti-viral adaptive immune responses, FAT-1 transgenic mice were used [3]. Spleen weights from uninfected wild type (WT) and FAT-1 mice were not significantly different. However, after 7 days of the infection with LCMV Amstrong, spleen weights from FAT-1 mice were significantly lower than those from WT mice (Figure 1A). Consistent with this result, the total numbers of splenocytes from FAT-1 mice were significantly less than those from WT mice after infection (Figure 1B). The spleen is an essential secondary lymphoid organ that generates adaptive immune responses against the infection with viruses including LCMV [29]. Thus, the reduction of splenocytes in FAT-1 mice could result in delayed clearance of the virus. To examine this possibility, we titrated the virus in the serum of WT and FAT-1 mice 7 days post-infection. Interestingly, virus titers were not significantly different between WT and FAT-1 mice (Figure 1C). These results indicated that the attenuated expansion of splenocytes in FAT-1 mice did not significantly affect the clearance of the virus.

### 2.2. Anti-Viral CD8^+^ T Cell Responses Were Suppressed in FAT-1 Mice

**Figure 2:**
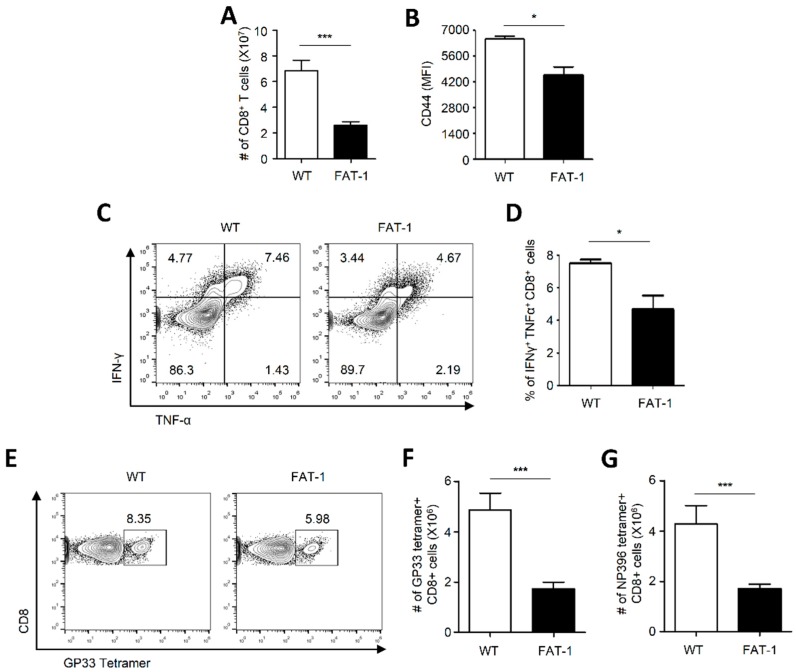
Anti-viral CD8^+^ T cell responses from spleen samples were suppressed in FAT-1 mice. WT and FAT-1 mice were infected with LCMV (Armstrong) for 7 days. (**A**) The number of CD3^+^/CD8^+^ cells from each spleen is shown. (**B**) The surface expression of CD44 on CD8^+^ T cells was analyzed using flow cytometry. (**C**,**D**) Splenocytes from WT and FAT-1 mice were stimulated with LCMV GP33 (1 µg/mL) peptide for 5 h followed by intracellular staining with anti-IFN-γ and TNF-α antibodies. The representative flow cytometry plots for expression of IFN-γ and TNF-α were shown (**C**). The frequency IFN-γ^+^ TNF-α^+^ CD8^+^ T cells are shown (**D**). (**E**) Splenocytes from WT and FAT-1 mice were stained with LCMV GP33 tetramer and the representative flow cytometry plots were shown. (**F**,**G**) The numbers (#) of LCMV GP33 (**F**) and NP396 (**G**) tetramer^+^ cells were graphed. The data represent the mean ± SEM (*n* = 4–8 mice per group). * *p* ≤ 0.05; *** *p* ≤ 0.001. The experiments were repeated at least three times with similar results.

Since CD8^+^ T cells are major effector immune cells against LCMV infection [30], we next compared anti-viral CD8^+^ T cell responses between WT and FAT-1 mice upon LCMV infection. To this end, FAT-1 and WT mice were infected with LCMV Armstrong for 7 days to analyze CD8^+^ T cell responses. Similar to splenocyte populations in Figure 1B, significantly less CD8^+^ T cells were observed in FAT-1 mice when compared to WT mice (Figure 2A). In addition, CD8^+^ T cells of FAT-1 mice expressed significantly lower levels of the activation marker CD44 than those of WT mice (Figure 2B). Further, we compared the ability of CD8^+^ T cells between WT and FAT-1 mice to produce anti-viral cytokines, IFN-γ, and TNF-α, in response to the LCMV epitope peptide glycoprotein_33–41_ (GP33). Frequencies of IFN^+^/TNF^+^ CD8^+^ T cells (Figure 2C,D) from FAT-1 mice were significantly diminished when compared to those of WT mice. Consistent with these results, lower frequency (Figure 2E) and number (Figure 2F) of GP33 tetramer^+^/CD8^+^ T cells in spleens of FAT-1 mice were observed than that in WT mice. We also found similar reduction of LCMV epitope peptide nucleoprotein_396–404_ (NP396) tetramer^+^/CD8^+^ T cells in spleens of FAT-1 mice as compared to those of WT mice (Figure 2G). These results collectively indicated that anti-viral CD8^+^ T cell responses were attenuated in FAT-1 mice.

### 2.3. Anti-Viral CD8^+^ T Cell Responses in Peripheral Blood of FAT-1 Mice Were Suppressed

**Figure 3:**
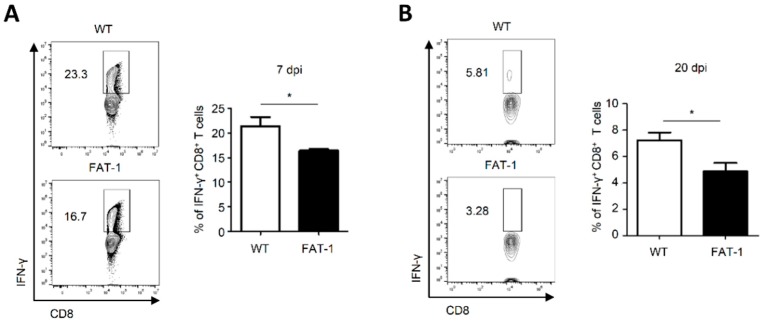
Anti-viral CD8^+^ T cell responses in peripheral blood were reduced in FAT-1 mice. WT and FAT-1 mice were infected with LCMV (Armstrong). Peripheral blood mononuclear cells were isolated from blood that was collected at 7 (**A**) or 20 (**B**) days post-infection. After stimulation with LCMV GP33 peptide, the expression of IFN-γ was analyzed using flow cytometry. The representative flow cytometry plots were shown (left panels) and the bar graph shows the percentage of IFN-γ^+^ CD8^+^ T cells (right panels). The data represent the mean ± SEM (*n* = 4–5 mice per group). * *p* ≤ 0.05. The data represent the results of at least three independent experiments.

We next examined whether reduced anti-viral CD8^+^ T cell responses are also observed in different compartments such as peripheral blood (PB) and liver. When compared to WT mice, FAT-1 mice showed significantly lower frequency of IFN-γ-producing CD8^+^ T cells in PB after exposure to LCMV GP33 7 dpi (Figure 3A). Intriguingly, the frequencies of IFN-γ-producing CD8^+^ T cells in PB between WT and FAT-1 mice showed the same trend at 20 dpi as they did for 7 dpi (Figure 3B). This indicates that impaired CD8^+^ T cell responses in FAT-1 mice are not transient. In addition, the number of LCMV NP396 in livers of FAT-1 mice was lower than that of WT mice (data not shown). These results were consistent with the data obtained from spleens (Figure 1). Together, these results suggested that attenuated anti-viral CD8^+^ T cell responses were observed not only in a lymphoid organ but also in other compartments and maintained even at 20 dpi.

### 2.4. Anti-Viral CD8^+^ T Cell Responses in FAT-1 Mice Were Affected by Extracellular Factors

**Figure 4:**
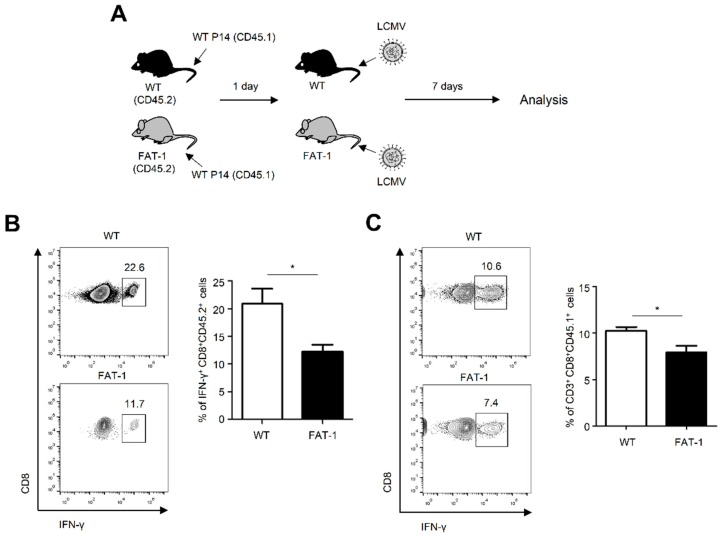
Wild type anti-viral CD8^+^ T cell responses were suppressed in FAT-1 mice. (**A**) P14 cells were adoptively transferred into WT and FAT-1 mice 1 day before infection with LCMV (Armstrong) for 7 days. (**B**,**C**) Splenocytes were stimulated with LCMV GP33 peptide for 5 h and intracellular staining occurred for IFN-γ. The representative flow cytometry plots for the expression of IFN-γ in CD45.2^+^CD8^+^ cells (**B**) or CD45.1^+^CD8^+^ cells (**C**) were shown (left panels). The graph shows the percentage of IFN-γ-producing cells (right panels). The data represent the mean ± SEM (*n* = 3 mice per group). * *p* ≤ 0.05. The data represent the results of at least three independent experiments.

Next, we sought to determine whether reduced anti-viral CD8^+^ T cell responses in FAT-1 mice were driven by environmental factors specific to FAT-1 mice. To this end, WT LCMV GP33 specific TCR transgenic CD8^+^/CD45.1^+^ T (P14) cells were adoptively transferred into CD45.1^−^/CD45.2^+^ WT and FAT-1 mice, followed by infection with LCMV for 7 days (Figure 4A). Consistent with our results in Figure 2, IFN-γ-producing endogenous CD8^+^ T cells (CD45.2^+^) in FAT-1 mice were significantly lower than those in WT mice in response to LCMV GP33 exposure (Figure 4B). Interestingly, the frequency of adoptively transferred exogenous WT P14 cells was also significantly diminished in FAT-1 mice when compared to WT mice (Figure 4C). Thus, these results indicated that factors specific to FAT-1 mice suppressed anti-viral CD8^+^ T cell responses.

### 2.5. Anti-Viral CD4^+^ T Cell Responses in FAT-1 Mice Were Impaired

**Figure 5:**
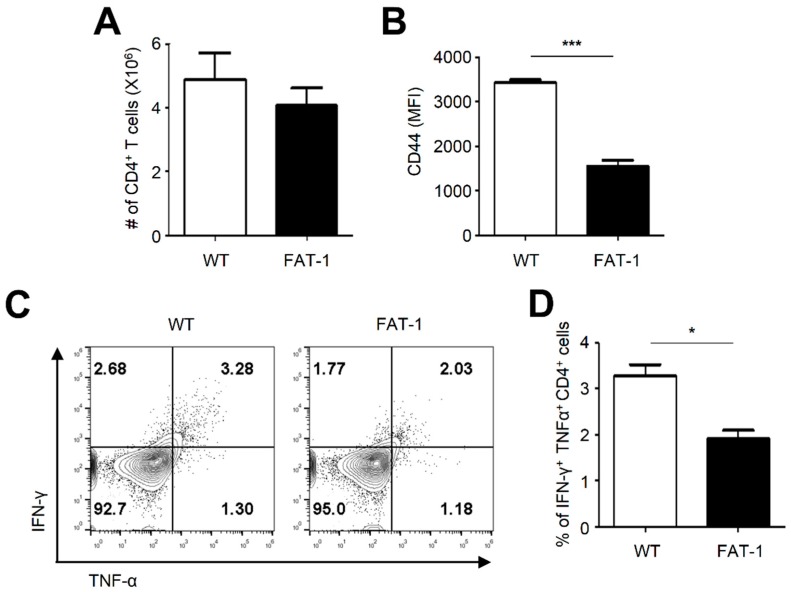
Anti-viral CD4^+^ T cell responses are diminished in FAT-1 mice. WT and FAT-1 mice were infected with LCMV (Armstrong) for 7 days. (**A**) The numbers of CD3^+^/CD4^+^ T cells harvested from spleens are shown. (**B**) The surface expression of CD44 on CD4^+^ T cells of WT and Fat-1 splenocytes was analyzed using flow cytometry. (**C**,**D**) Splenocytes were incubated with µg/mL of LCMV GP61 peptide for 5 h, and intracellular staining for IFN-γ and TNF-α was performed. The representative flow cytometry plots were shown (**C**). The percentage of IFN-γ^+^ TNF-α^+^ CD4^+^ T cells was analyzed using flow cytometry (**D**). The data represent the mean ± SEM (*n* = 4 mice per group). * *p* ≤ 0.05; *** *p* ≤ 0.001. The data represent the results of at least three independent experiments.

CD4^+^ T cells are critical immune effectors to orchestrate immune responses against infections. This led us to hypothesize that anti-viral CD4^+^ T cell responses are also hampered in FAT-1 mice. To test this, WT and FAT-1 mice were infected with LCMV for 7 days, and anti-viral CD4^+^ T cell responses were analyzed. Although the number of splenic CD4^+^ T cells was not significantly different between WT and FAT-1 mice (Figure 5A), the expression level of CD44 on splenic CD4^+^ T cells in FAT-1 mice was significantly lower than that of WT mice (Figure 5B). Moreover, when splenocytes were stimulated with the LCMV epitope peptide glycoprotein_61–80_ (GP61) (Figure 5C,D), frequencies of IFN-γ^+^/TNF^+^ CD4^+^ T cells (Figure 5D) from FAT-1 mice was significantly lower than that of WT mice. These results showed that anti-viral CD4^+^ T cell responses were impeded in FAT-1 mice.

### 2.6. Anti-Viral CD8^+^ T Cells Carrying the Fat-1 Gene Exhibited a Decreased Capability to Proliferate and Produce Inflammatory Cytokines against LCMV Infection

**Figure 6:**
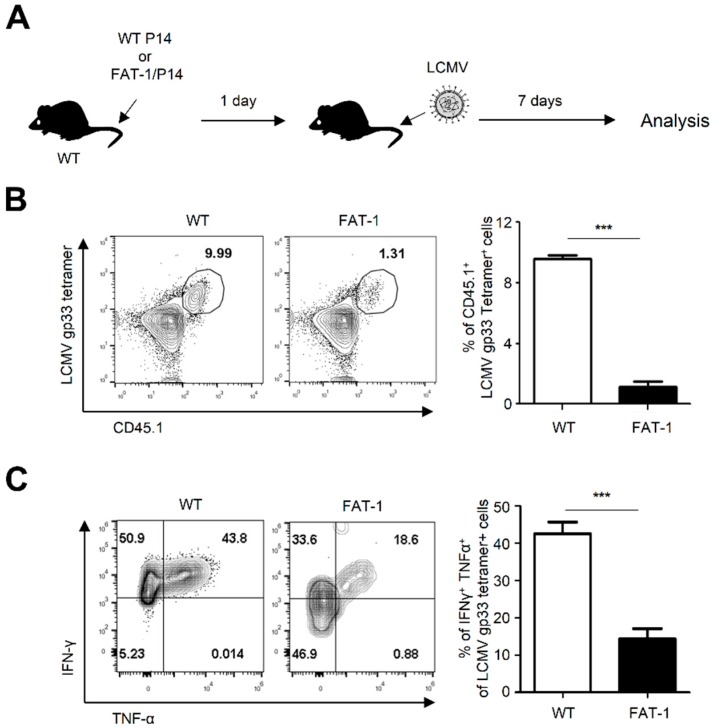
FAT-1/P14 CD8^+^ T cells exhibited less proliferation and cytokine production than WT P14 cells. (**A**) WT P14 and FAT-1/P14 cells were adoptively transferred into naïve WT mice, followed by infection with LCMV (Armstrong) for 7 days. (**B**) Splenocytes were stained LCMV GP33 tetramer/CD45.1 antibody and analyzed by flow cytometry. (**C**) Splenocytes were stimulated with LCMV GP33 peptide to analyze the numbers of IFN-γ- and TNF-α-producing CD8^+^ T cells using flow cytometry. The representative flow cytometry plots were shown in left panels and the bar graph indicates the percentages of IFN-γ- and TNF-α-producing CD8^+^ T cells (right panels). The data represent the mean ± SEM (*n* = 5 mice per group). *** *p* ≤ 0.001. The data represent the results of at least three independent experiments.

Although extracellular factors in FAT-1 mice were shown to regulate anti-viral CD8^+^ T cells responses (Figure 4), the possibility that cell-intrinsic *n*-3 PUFAs directly affect CD8^+^ T cell responses against LCMV infection cannot be excluded. To examine this, we generated P14 T cells carrying the *fat-1* gene (FAT-1/P14) by crossing P14 mice with FAT-1 mice. These FAT-1/P14 or WT P14 cells were adoptively transferred into WT mice, followed by infection with LCMV (Figure 6A). Seven dpi, the expansion and activation of the transferred cells, were analyzed by flow cytometry. Remarkably, the expansion of FAT-1/P14 cells (1.17 ± 0.25%) was substantially hampered when compared to WT P14 cells (8.40 ± 0.36%) (Figure 6B). Furthermore, when these cells were stimulated with LCMV GP33, significantly lower expression levels of IFN-γ and TNF-α were observed (Figure 6C). These results suggested that cell-intrinsic *n*-3 PUFAs dampened CD8^+^ T cell activation upon LCMV infection.

## 3. Discussion

The activation of anti-viral adaptive immune responses is crucial in fighting viral infections. However, unnecessary immune responses such as cytokine storm can cause tissue injury and death. Thus, controlling excessive inflammation without affecting viral clearance efficacy of immune cells is considered important in the treatment of viral infection. In this study, we investigated the possible implication of *n*-3 PUFAs in anti-viral immune responses. We found that anti-viral CD8^+^ T cell responses were significantly decreased in *n*-3 PUFA-rich mice along with the successful elimination of viruses. Importantly, the cell-intrinsic expression of *n*-3 PUFAs in CD8^+^ T cells suppressed their activation against viral infection. Since CD8^+^ T cells are major contributors for virus infection-mediated immunopathology, *n*-3 PUFAs could possibly be used to alleviate virus infection-mediated immunopathology.

In several previous studies, *n*-3 PUFAs were shown to display anti-inflammatory effects on diverse inflammatory diseases such as asthma and arthritis [31]. While many studies have focused on their regulation of CD4^+^ T cell activation, the effect of *n*-3 PUFAs on anti-viral CD8^+^ T cells was not well known. In this study, we generated P14 cells expressing the *fat-1* gene (FAT-1/P14) by crossing FAT-1 mice with P14 mice. The experiments using these cells could provide direct evidence that cell-intrinsic *n*-3 PUFAs suppressed anti-viral CD8^+^ T cell responses (Figure 6). Several mechanisms could be involved in the inhibitory activity of *n*-3 PUFAs on anti-viral CD8^+^ T cell responses. First, receptor-ligand interactions such as TCR-pMHC and costimulatory receptor/ligand interactions between CD8^+^ T cells and virally infected target cells might be affected by membrane fluidity and lipid rafts. Since *n*-3 PUFAs are known to regulate plasma membrane fluidity and lipid raft formation [32,33,34], membrane-incorporated *n*-3 PUFAs might interfere with efficient interaction between receptor and ligand interaction. Second, intracellular *n*-3 PUFAs or their derivatives could serve as signaling cascade regulators responsible for activation of anti-viral immune responses. For example, *n*-3 PUFAs were shown to alter IL-6/Stat3 pathway and displace the Src family kinases Lck and Fyn [35,36]. Thus, *n*-3 PUFAs possibly modify several intracellular signaling pathways critical for anti-viral CD8^+^ T cell activation. These possible mechanisms require further investigation.

Chronic viral infections such as HIV, hepatitis C virus, and hepatitis B virus are known to be associated with the exhaustion of anti-viral CD8^+^ T cells [37,38]. Thus, prevention of T cell exhaustion is considered as a promising therapeutic strategy to treat chronic infections. T cell exhaustion is known to be caused by exposure to persistent antigens and inflammatory signals [39]. We demonstrated that endogenous *n*-3 PUFAs attenuated the activation of CD8^+^ T cells in response to viral antigen. Thus, *n*-3 PUFAs possibly interfere with the process of T cell exhaustion, which could be a novel therapeutic strategy to treat chronic infection. Further investigation will be needed for this hypothesis.

Our data indicated that cell-intrinsic *n*-3 PUFAs suppressed anti-viral CD8^+^ T cell responses (Figure 6). We also interestingly found that, when WT P14 cells were adoptively transferred into FAT-1 mice, their activation was decreased (Figure 4). This indicates that anti-viral CD8^+^ T cell responses were negatively regulated by environmental factors specific to FAT-1 mice. It is possible that some of the abundant extracellular *n*-3 PUFAs in FAT-1 mice are incorporated into adoptively transferred WT P14 cells, which might result in the reduced activation of the cells. Another possibility is that impaired activation of CD4^+^ T cells in FAT-1 mice (Figure 5) leads to reduced secretion of inflammatory cytokines that aid in the enhancement of CD8^+^ T cell activation.

Collectively, our results support that *n*-3 PUFAs serve as anti-inflammatory mediators on anti-viral CD8^+^ T cell responses. Thus, using *n*-3 PUFAs as a dietary supplement could help aid in the alleviation of immunopathology seen during diverse viral infections.

## 4. Materials and Methods

### 4.1. Mice

C57BL/6 (CD45.1^−^/CD45.2^+^), C57BL/6-CD45.1^+^Db GP33–41 (P14) T cell receptor (TCR) transgenic (tg) (a gift from Dr. Sang-Jun Ha of Yonsei University, Seoul, Korea) (CD45.1^+^/CD45.2^−^), and C57BL/6-FAT1 tg (kindly provided by Dr JX Kang of Harvard Medical School, Boston, USA) (CD45.1^−^/CD45.2^+^), and C57BL/6-P14-FAT-1 (CD45.1^+^/CD45.2^−^) mice were used. C57BL/6-P14-FAT-1 mice were generated by crossing FAT-1 mice with P14 mice. Briefly, P14 mice were crossed with FAT-1 mice to generate F1 mice. The F1 mice were then crossed with each other and the resulting F2 mice were screened for the presence of *fat-1* gene and P14 transgenic receptor. All mice were bred and maintained in a closed breeding facility and transferred to an animal biosafety level 2 (ABSL2) facility of the Korea Zoonosis Research Institute (KoZRI) for LCMV infection. All experiments were performed according to the protocol approved by the Institutional Animal Care and Use Committee at Chonbuk National University (CBNU 2019-013).

### 4.2. Virus and Infection

LCMV Armstrong strain was kindly provided by Dr. Sang-Jun Ha of Yonsei University. LCMV Armstrong was propagated in baby hamster kidney cells [40]. For the in vivo infection study, mice were infected by intraperitoneal (i.p.) administration of 2 × 10^5^ focus-forming units (FFU) of LCMV. Uninfected mice were used in all experiments shown in figures as background controls.

### 4.3. Reagents and Antibodies

For splenocytes culture, RPMI medium supplemented with penicillin/streptomycin (Wellgene, Seoul, Korea) and 10% fetal bovine serum (Hyclone, Pittsburgh, PA, USA) were used. Anti-mouse CD3-APC Cy7, CD8-FITC, CD45.1-PerCP Cy5.5, CD44-PE, and IFN-γ-FITC were purchased from Tonbo Bioscienses (San Diego, CA, USA). Anti-mouse TNF-α-PE and Cy7 APC-conjugated streptavidins were purchased from Biolegend (San Diego, CA, USA).

### 4.4. Determination of LCMV Titers

To titrate the LCMV (Armstrong), focus-forming assay was performed as published previously [41]. Vero cells were plated on 24 well plates on day before the assay. The cells were then incubated with diluted serum collected from mice infected with the virus for 1 hr. Cells were incubated with 10% FBS-DMEM with 1% methylcellulose. Two days later, following fixation and permeabilization, cells were stained with anti-LCMV antibody VL4 (Biocell, West Lebanon, NH, USA) and goat anti-Rat IgG (Thermo Fisher Scientific, Waltham, MA, USA). Foci were developed by DAB (Thermo Fisher Scientific, Waltham, MA, USA) solution to determine viral titers.

### 4.5. Isolation of Splenocytes

Spleens that were harvested from the mice were mashed in 70 µm cell strainers (SPL). Red blood cells were lysed with ACK lysis buffer (Thermo Fisher Scientific, Waltham, MA, USA) for 4 min. After washing twice with PBS, splenocytes were resuspended in RPMI-1640 containing 10% FBS and penicillin/streptomycin for subsequent experiments.

### 4.6. Intracellular Cytokine Staining

Splenocytes were harvested from mice 7 days post-infection. Harvested splenocytes were incubated for 5 h in RPMI-1640 medium containing µg/mL GP33 (Genescript, Piscataway, NJ, USA) and 2 µg/mL brefeldin A (Sigma, St. Louis, MO, USA). Cells were stained with anti-mouse CD3-APC Cy7, CD8-PE, CD45.1-PerCP Cy5.5, and LCMV GP33 Tetramer-APC and permeabilized using Foxp3/Transcription Factor Staining Buffer Kit according to the manufacturer’s protocol. After permeabilization, cytokines were detected using anti-mouse IFN-γ-FITC and TNF-α-PE Cy7 (Biolegend, San Diego, CA, USA).

### 4.7. Flow Cytometric Analysis

WT and FAT-1 P14 CD8^+^ T cells were transferred into WT mice and infected with LCMV Armstrong one day later. Seven days post-infection, the cells were harvested and resuspended in flow cytometry buffer (BSA 1%, EDTA 2 mM and sodium azide 0.1%). For detection of the cell surface marker, cells were stained with the following antibodies: anti-mouse CD3-APC Cy7, CD8-FITC, CD45.1-PerCP Cy5.5, and CD44-PE. Data were acquired with the Attune^TM^ NxT Acoustic Focusing Cytometer (Thermo Fisher Scientific, Waltham, MA, USA) and analyzed using a Flowjo software (BD Biosciences, San Diego, CA, USA).

### 4.8. Statistical Analysis

Error bars indicate the standard error of the mean (SEM), and mean values were compared using Student’s *t*-tests. All experiments were repeated independently at least three times.
